# Investigation of Dipolar Response of the Hydrated Hen-Egg White Lysozyme Complex under Externally Applied Electric Fields: Insights from Non-equilibrium Molecular Dynamics

**DOI:** 10.1021/acs.jpcb.1c07096

**Published:** 2022-01-21

**Authors:** HaoLun Wu, Mohammad Reza Ghaani, Prithwish K. Nandi, Niall J. English

**Affiliations:** † School of Chemical & Bioprocess Engineering, University College Dublin, Belfield, Dublin 4, Ireland; ‡ Irish Centre for High-End Computing, Trinity Enterprise Centre, Pearse Street, Dublin 2, Ireland

## Abstract

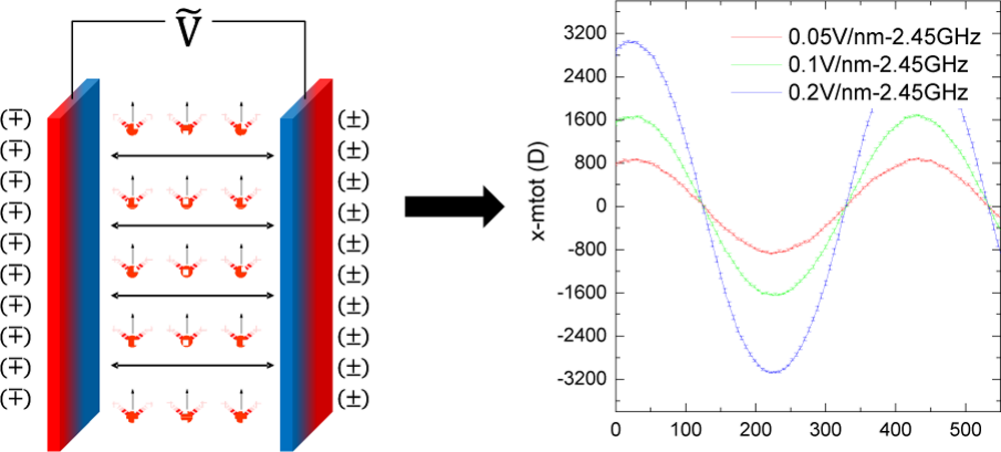

Given its ubiquitous presence in the environment of bio-macromolecules, water is well known to play a fundamental part in biological activity, often as a regulating agent. In parallel, with increasing attention focused on the potential damage of microwave-frequency radiation exposure to human health, the effects of extraneous electric and electromagnetic (e/m) fields on water shells surrounding proteins, and, indeed, biomolecules themselves, are becoming a particularly pertinent issue. In this study, non-equilibrium molecular dynamics simulations of hydrated hen-egg white lysozyme have been performed in both the absence and presence of external electric fields of varying intensity (0.005–0.02 V/Å) and frequency (static, *i.e.*, zero-frequency, together with oscillating fields of 2.45–100 GHz). By comparing the effect of different electric-field conditions on both the protein’s and surrounding hydration layer’s dipole moments and their underlying relaxation dynamics, clear and evident non-thermal field effects were observed on the dipolar response of both the protein and hydration layer. This occurred primarily as a consequence of the protein’s dipolar alignment with the external field and increased with the growth of field intensity. In addition, it was found that the lag time of dipolar response to the applied field itself, for both the protein and the first hydration sub-shell (*i.e.*, directly adsorbed layer), under oscillating fields is longer than that in both the second hydration sub-layer and bulk water, owing to strong direct protein–water adsorption. In that respect, we also probe and discuss the effect of protein–water hydrogen bonds, dissecting the subtleties of “bio-water” dipolar response.

## 1 Introduction

The interaction between water and proteins is of pivotal influence on the properties and behavior of the proteins—indeed, their structure, together with biological function and biophysical “personality.” This includes, inter alia, chain folding, conformational stability, internal dynamics, and enzyme catalysis,^1−3^ in living organisms. Generally, proteins are surrounded by water molecules comprising a dynamical hydration layer, which is significantly different to bulk-like or bulk solvent water vis-à-vis its structural and dynamical properties, and the number of these type of water molecules depends on the requirement for biological functionality.^4^ Thus, proteins and their hydration layer must be regarded instead as a complex entity, mutually inter-dependent and interacting, which determine and serve to regulate organisms’ biological activities *in vivo*.^5−7^

The physical structures originating from hydrated water can essentially be sub-divided into their mechanistic origins from the behavior of three sub-layers.^8^ The first layer is composed of directly bonded water restrained by charged or polar residues inside the protein. The second layer is hydration water that interacts directly with protein surfaces by forming hydrogen bonds therewith. The final outermost sub-shell consists of water molecules under the electrostatic influence of the proteins, which is just beyond the van der Waals contact distance; this is typically dubbed more bulk-like water, although it shows characteristics intermediate between true bulk-like water and directly adsorbed sub-layers. Due to the complex surfaces of proteins and disordered motion of water molecules,^3^ water molecules only remain shortly in the hydration layer, and the underlying dynamical processes of the hydration water often last over the picosecond time scale.^9−11^ However, it is challenging to elaborate the mechanism of protein–water interaction over long time scales, due to the inherent difficulty in observing the interactive behavior of the water–protein complex. Therefore, understanding the subtleties of dynamical coupling governing the water–protein “bio-complex” is critical to characterize its three-dimensional structure and understand the protein’s underlying dynamics.

Although the heterogeneous nature of proteins themselves, and their “patchwork” surfaces, is reflected in the varied and non-uniform network of hydration water atop, this level of protein–water coupling is relatively weak for a small protein, albeit magnified for larger ones, which exert a greater local electric field in their immediate hydration-layer milieux. In any event, the structure and dynamics of water molecules surrounding a complex protein are heterogeneous in nature, not only reflecting the varied topography of the underlying protein surface but also interacting with and regulating this. Since water molecules behave differently at a protein surface to their bulk state, especially near the more pronounced hydrophobic and hydrophilic areas,^5,12^ it is a considerable challenge to explore their interactions (either with each other and the underlying protein) at the atomistic level. Indeed, this inherent difficulty has motivated a great deal of activity and raised widespread debate, particularly about whether water or protein has ultimately the more dominant regulatory role in dynamical coupling.^13,14^ In any event, based on neutron scattering and nuclear magnetic resonance (NMR), the influence of proteins, such as lysozyme and myoglobin, on the dynamics of their hydration water can be studied experimentally.^15^ Dielectric spectroscopy is also a useful tool and has been applied to identify different types of hen-egg white lysozyme (HEWL) hydration water molecules based on the distribution of δ and β dispersion.^16^ Focusing on the retardation of water molecules’ translational and rotational motion in hydration layers, Sterpone *et al.*(17) established with acuity a jump-reorientation model, in which the topological excluded-volume factor of the local protein geometry and the free energetic factor of hydrogen bonding control the inherently slower dynamics of the hydration layer. Indeed, analysis of a fluorescence shift^11^ serves to rationalize the extremely slow decay of hydration dynamics by a solvent-polarization mechanism: conformational fluctuations of a polarized protein “update” the status of adsorbed and surrounding water molecules enveloping its surface. In any event, it is clear that the hydrogen bonding and local intrinsic electric-field conditions (as will be quantified and discussed below) atop the baroque protein surface offer a venue for dynamical protein–water coupling and rich, subtle interplay.

One open question in contemporary biophysics,^12−14^ also of great topical importance to health and communications, is in probing and unpicking the effect of extraneous and external electric fields on biological systems—with proteins as an important case in point. Despite the response of biological macromolecules, such as proteins, to electromagnetic (e/m) fields typically giving rise to thermal heat generation by e/m-wave absorption (due to the molecular friction generated by oscillating-dipole alignments), it is essential to explore the precise mode of action of non-thermal electric-field effects,^18^ which are less understood. Indeed, these athermal effects can alter molecules’ conformations by exciting its vibrational modes. Due to the clear risk and basis that e/m fields can affect the structural and functional stability of proteins, scrutiny of the effects of far-infrared and microwave fields on macromolecules has attracted widespread attention with the order-of-picosecond periods, affecting the underlying macromolecular relaxation processes of similar time scales;^18−24^ this is especially so in respect of possible effects on human diseases related to protein denaturation, as well as in exploring its potential clinical applications in protein engineering and medicine.^25,26^

Given that the fundamental mechanistic mode of action of molecular-system response to electric fields, when considering dipolar moieties and species, such as water and proteins, lies in their extent of dipole alignment from the applied field’s torque, in addition to how faithfully and quickly the system can “echo” and track the applied field, whether static or oscillating (*e.g.*, e/m), it is clear that studying how dipolar properties are altered becomes highly relevant. In fact, theory and application about the interfacial polarization of bio-macromolecules or cells as whole, such as dielectrophoresis, have shown considerable potential in the field of biology and medicine.^27−29^ Intriguingly, the question of how static and e/m electric fields affect and manipulate the behavior of hydrated proteins and their hydration layers and affect dynamical coupling has been studied very little, although Nandi *et al.*(30) made some important mechanistic progress in quantifying translational motion and diffusivity characteristics of the protein and its aqueous shell. Even so, there has been no study of the dipolar response of hydration layers, and, indeed, of the protein itself in that particular context. This is an important lacuna in the literature, and indeed, an important open question in its own right. Indeed, for such prototypical examples of dynamical systems like protein–water complexes, investigating the non-equilibrium, field-induced dipolar response of proteins and their local hydration water can help us understand more deeply the general structural and dynamical behavior adopted by hydrated proteins in external e/m fields.

Molecular dynamics (MD) has been used widely to explore, inter alia, the role of flexibility in ligand binding, to study the rapid solvation of the electron-transfer state in photosynthesis,^31^ to determine protein structures from NMR, and to calculate the free-energy changes resulting from mutations in proteins.^32^ For reproducing dielectric properties of a protein and its solvent, practicability of MD simulation has been verified by many previous studies.^33−37^ However, comparatively few studies have focused on the non-thermal effects of e/m fields on proteins—most especially on the induced conformational changes in lysozyme, amyloid fibrils, and trans-membrane proteins.^38−41^ In some of our previous studies,^42,43^ taking HEWL as a test case and employing non-equilibrium MDs in externally applied electric fields, we showed that the secondary protein structures are markedly perturbed by intense fields at 0.05–0.15 V Å^–1^, for both static and oscillating fields (2.45–500 GHz), leading to accelerated incipient denaturation. To explore the effect of e/m fields on a protein’s hydration layer, English and Mooney^43^ commented briefly on dipolar orientations of water in the immediate solvation layer of HEWL, and Todorova *et al.*(24) on hydration-layer water molecules’ dipolar-orientation kinetics around amyloid fibrils in e/m fields. However, it less complete in terms of biophysics to define a single sub-shell, quantifying the full response of the hydration layer to e/m fields because water molecules in the multi-layered hydration shell present large inherent oscillations in their polarizability.^44^ Thus, the exploration of the dipole response of water molecules in specific and well-defined hydration layers is essential in order to arrive at a more complete dipole-response insights.

In this study, bearing in mind this lack of dipolar-response characterization, classical MD simulations was used to investigate the non-thermal effect of e/m fields on a representative protein and its hydration layer. We focus on the local dipolar susceptibility of wild-type HEWL and the surrounding water molecules in different hydration sub-shells. Despite e/m fields also promoting kinetics of picosecond phenomena, such as IR phenomena, as previously mentioned, we focus in the present work on nanosecond time scales to gauge the interaction between the protein and the first (directly adsorbed) hydration layer on these inherent nanosecond-scale dipole-response and rearrangement dynamics.^35,45,46^ The use of molecular simulation for the purpose of dipole-response tracking is especially important^47−50^ since such shifting dipole moment dynamics over nanosecond time scales cannot be observed directly by experiments;^16^ non-equilibrium MD, in applied external fields, has the extra advantage of being able to simulate directly the dipolar-perturbation repose of the system, with its field-altered geometry and dynamical properties.^38−40^ Specifically, in the present work, we calculate the dipole moment and corresponding autocorrelation function (ACF) of HEWL itself and its hydration layer under zero-field, oscillating-field, and static-field conditions. The lag time of the protein and its hydration layer under the oscillating field were also studied to evaluate the effect of field’s frequency on the hydrated protein and surrounding water molecules.

## 2 Methods

The simulations were performed using modified version of the GROMACS-2018^51^ MD-simulation package featuring the AMBER99SB^52^ force-field and TIP4P/2005^53^ potential models for HEWL and water, respectively, owing to their good suitability for globular proteins.^54^ HEWL is a small globular protein with a molecular mass of 14,320 Da and triclinic wild-type, namely, the 2LZT PDB crystal structure, and is a good representative prototype for the typical mixture of hydrophobic and hydrophilic interactions typically encountered in such types of globular proteins.

In order to simulate the actual state of solvated proteins (reducing the influence of ions,^55,56^ and serving to represent the field effect on the protein itself), formal charges were chosen appropriate to pH 7, resulting in a total charge of +8e for this protein. A corresponding number of Cl counterions were placed throughout the solvent, such that the overall system was electroneutral. To avoid that protein atoms were lying within less than around 10 Å from the edge of the simulation box, the protein was placed at the center of a rectangular periodic box with (*x*, *y*, *z*) dimensions of 159.2, 55.2, and 61.7 Å, respectively, in the laboratory Cartesian frame of the original structure, with 17,486 molecules of water surrounding the protein structure.

Before MD was begun, energy minimization of the system was carried out with a composite protocol of the steepest descent, conjugate gradient, and truncated Newton steps. The system was equilibrated for a total time of 200 ps under *NVT* conditions and 500 ps under *NPT*, where temperature control was imposed using a velocity-rescaling approach with a stochastic term, ensuring proper canonical-ensemble sampling and featuring a time constant of 0.1 ps and a reference pressure of 1 atm.^57^ For *NPT* conditions, these were maintained throughout the entire simulation using the Parrinello–Rahman method with a time constant of 2 ps, as well as long-range dispersion corrections for energy and pressure. In terms of bond interactions, the LINear Constraint Solver (LINCS) method was applied to handle holonomic constraints, while long-range electrostatic interactions were handled by the smooth particle-mesh Ewald method—with a cut off and integration time step of 10 Å and 2 fs, separately.^57^ For field-exposed protein solution, vacuum boundary conditions were used in the Ewald summation to obtain realistic dielectric response.^58^

**Figure 1:**
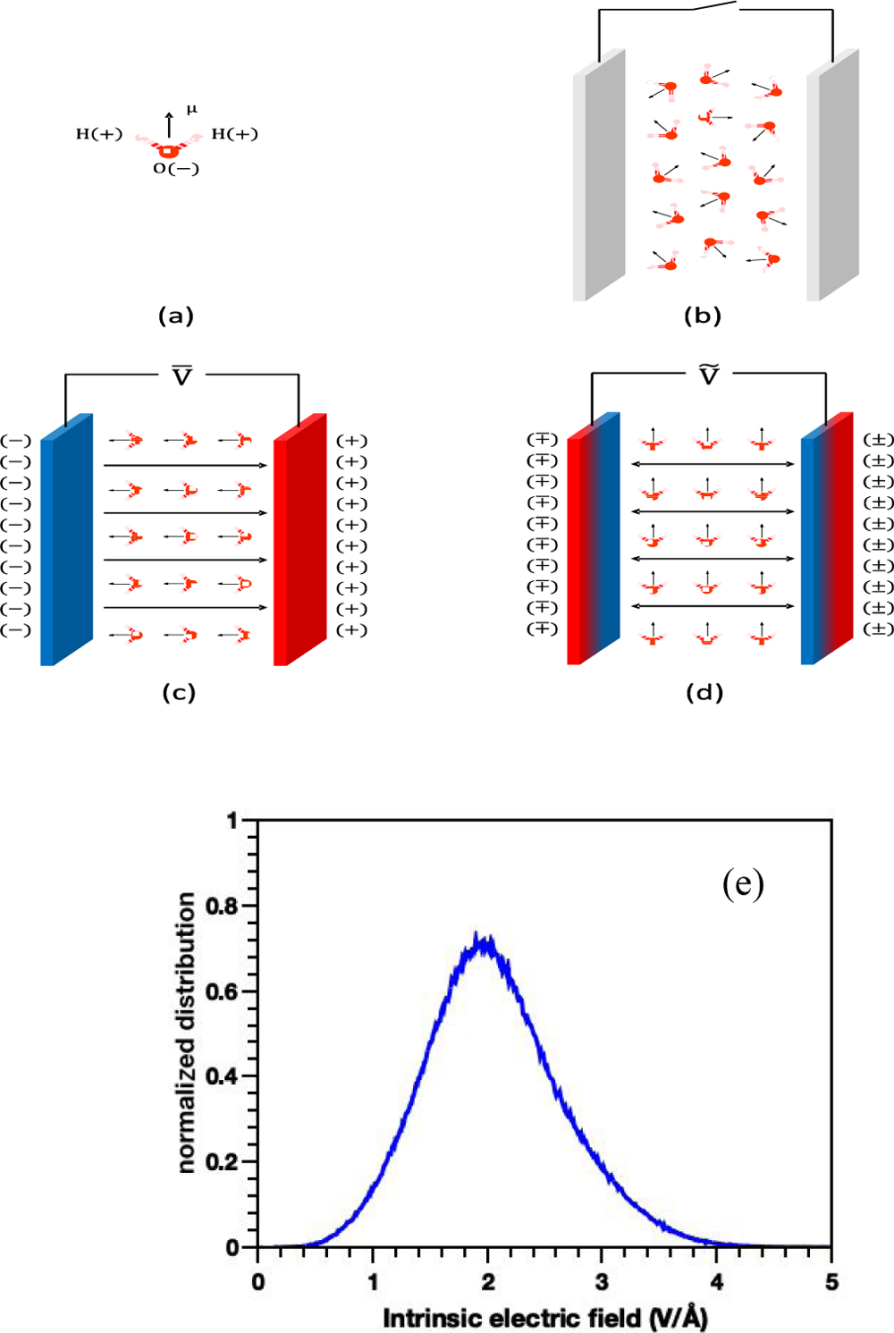
Polarization of the water molecules in a “thought experiment.” (a) Water molecule’s dipole moment, and (b) typical random orientation in the absence of any applied field, with (c) and (d) showing dipole-alignment characteristics of water molecules under external electric fields. (e) Intrinsic electric-field probability distribution in HEWL hydration-layer molecules at ambient temperature (in the absence of applied field).

Uniform external static and e/m fields were applied with the electric component *E* acting along the laboratory *x*-direction *E*(*t*) = *E*_max_ cos(ω*t*)*k*, as described in previous studies.
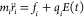
1where *q*_*i*_ denotes the charge and *f*_*i*_ denotes the force on site *i* due to the intermolecular potential. Classical mechanics was used for the treatment of the e/m absorption since the experimental spectrum of liquid water is continuous in the low-frequency microwave region.^59^ Conceptually, for a water molecule, the oxygen atom has a partial negative charge since oxygen has a higher electronegativity than hydrogen (*cf.*Figure 1a). Carrying out a “thought experiment,” under a static electric field (*cf.*Figure 1b), water molecules are polarized, and the field’s toque acting on the molecule’s dipole leads to dipolar orientation in the direction of the field, provided that the applied-field torque is sufficient to overcome local hydrogen-bonding interactions with neighboring molecules in condensed states; such an alignment is depicted in Figure 1c, which serves to strengthen the hydrogen-bond network. However, under oscillating fields (*cf.*Figure 1d), continual and periodic (*i.e.*, cosine) dipolar reorientation occurs due to cycling torque-induced rotation of molecules. In any event, this “concept-diagram” in Figure 1 is illustrative, in the sense that TIP4P/2005 is a fixed-charge potential, and there is no inherent polarizability therein, although it is compatible with various biomolecule force fields;^34^ still, the basic mechanistic features of the qualitatively different types of field orientation are evident for both static and oscillating fields.

In our Non-Equilibrium Molecular Dynamics (NEMD) simulations, the applied e/m fields were of frequency ϑ = 2.45, 10, and 100 GHz (corresponding, respectively, to periods of 408, 100, and 10 ps) and of rms intensity *E*_rms_ = 0.005, 0.01, and 0.02 V/Å—with the same rms intensities acting as constant field strengths for static electric fields. These are of the order of (up to) 1% of intrinsic electric fields in the HEWL hydration layer (*cf.*Figure 1e). In particular, the 0.005 V/Å intensity is lower than that of the experimental dielectric-breakdown threshold (about 0.006 V/Å).^24^ In any event, although the intensity of the fields applied in this study is about 2 or 3 orders of magnitude larger than those applied in the industry and experiment,^25^ previous NEMD simulations of microwave and IR field effects on water^59−61^ and other materials^12−15,17^ have shown that it is necessary to apply e/m field intensities of the order of 0.01 V/Å to observe tangible effects within limited nanosecond time scales. As mentioned in our previous work,^43^ an rms intensity of 0.01 V/Å led to statistically indistinguishable changes in HEWL mutants’ root-mean-square deviations (rmsds) or gross dipolar alignments over 25 ns vis-a-vis zero-field conditions in either static or 2.45 GHz e/m fields; this observation is in accordance with gas-phase simulations of polyalanines in static 0.01 V/Å electric fields performed by Calvo and Dugourd.^62^ Indeed, previous analysis has indicated that the rms intensity exhibits a linear-type response to dipole alignment up to about 0.05 V/Å,^61^ a behavior we would also expect in the current work’s analysis. Nevertheless, despite the linear-response régime being expected to apply in the present study, the use of non-equilibrium MD simulation is still needed to witness the “cause and effect” of system response to partial dipolar alignment. The external fields were applied in conjunction with *NPT* coupling and are referred to as non-equilibrium *NPT* (N*NPT*) simulations.^60^ Once the system was stabilized thermally, a production run of 100 ns was carried out in electric fields under each N*NPT* condition (*i.e.*, both static and oscillating fields), as well as in the 100 ns *NPT* case under zero-field conditions. In all cases, trajectories were sampled every 1 ps for analysis. Due to commensurately longer simulation times in this work of up to 100 versus 1–2 ns, the field-intensity range in this study was approximately three times lower than that used previously for higher-frequency e/m and far-infrared fields’ 50–500 GHz studies on wild-type HEWL.^42^

We also wished to study systematically the effect of salt-ionic concentrations and simulation-box size (and shape) on HEWL behavior. In these cases, to set up the varying simulation-box size and ionic concentration, we first ran simulations featuring an elongated (*i.e.*, now-cubic) box (*i.e.*, 159.2^3^ Å) and a higher (150 mM) salt concentration, for which there were 26 cases: (1 + 3 + 3 × 3) × 2, that is, zero field, static field (3), and oscillating field (3 × 3) for each simulation-box size and ionic concentration. Again, each trajectory was of 100 ns in total duration, and all of them used the same equilibrium (*NVT* and *NPT*) parameters, as shown above. It can be seen that the box size and salt concentration did not affect the thermal motion of HEWL (*cf.* Figure S1a, Supporting Information) and dipolar orientation of HEWL and water molecules (*cf.*Figures S2a and S3a). However, in the 0.2 V/nm static field, there were significant denaturation (*cf.*Figure S1d) and depolarization of HEWL (*cf.*Figure S2d) after 50 ns if we added too many extra ions in the rectangular box. This is discussed further in the next section.

During the 100 ns “production” simulations, water molecular would “flit” back and forth between different hydration-layer sub-shells, and, therefore, the whole trajectories were divided into about 45 overlapping 10 ns time-sampling periods, during which the composition of water molecules in the sub-shells was stable for about 90% of this time segment. To guarantee a sufficiently long residence time of water molecular and the same electric-field status, the beginning time point between each segment was shifted by the integer-multiple-time of the e/m-field period.

## 3 Results and Discussion

**Figure 2:**
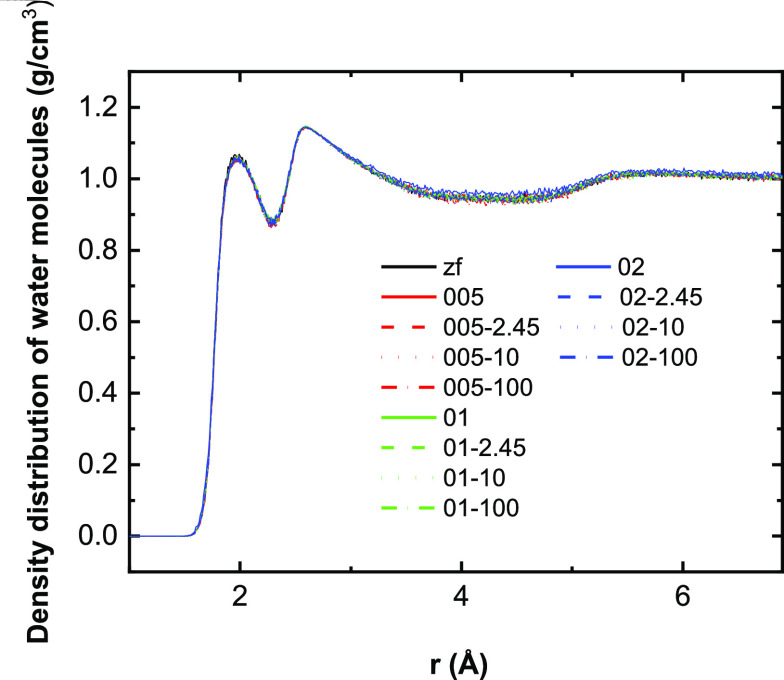
Density distribution of water molecules in the surroundings of the HEWL surface.

In order to quantify the changes in translational dynamics of the protein and its hydration layer, under external fields, the primary step is the identification of the hydration-layer sub-shells for HEWL, which has an irregular shape, *ipso facto*, as a globular protein. The accurate estimation of the hydration sub-shells’ volume is essential to the normalization of the spatial hydration-density distribution enveloping the protein. In the present study, state-of-the-art Voronoi-cell analysis^63^ was used to compute the volume of hydration sub-shells around HEWL, in which each individual water molecule’s contribution is counted to the protein–water complex’s total hydration-shell volume. Figure 2 shows the density distribution of the water molecules from the protein surface under different external-field conditions, and it is obvious there are two distinct hydration sub-layers with marked density separation. The first hydration layer presents within 2.25 Å from the surface of HEWL, where there is a less prominent minima location marking the second hydration sub-layer. From a distance of 6 Å from the protein surface, the density of water approaches the normal bulk-water density of 1 g/cm^3^, meaning that molecules are akin to bulk water, at least in their density. Although plotted under field conditions with quite different intensity and frequency, the density distribution of water molecules does not change significantly. According to Marracino *et al.*,^32^ applied fields with 0.5 V/Å intensity can only cause less than 5% variations in water density around ions statistically, which is much stronger than the highest intensity of 0.02 V/Å in this study. Similarly, water molecules in the first hydration layer have the same contact with the charged residues at the protein surface, and “further-out” water molecules are affected more by water–water interactions. Moreover, under the relatively low-intensity fields applied in the present study, these are not sufficient to give rise to visible conformational changes on the protein, meaning that there is no change in the number of charged residues and solvent-accessible surface area.^57^ Thus, the application of the present external electric fields has relatively little effect on the interaction type of water molecules surrounding the protein.

**Figure 3:**
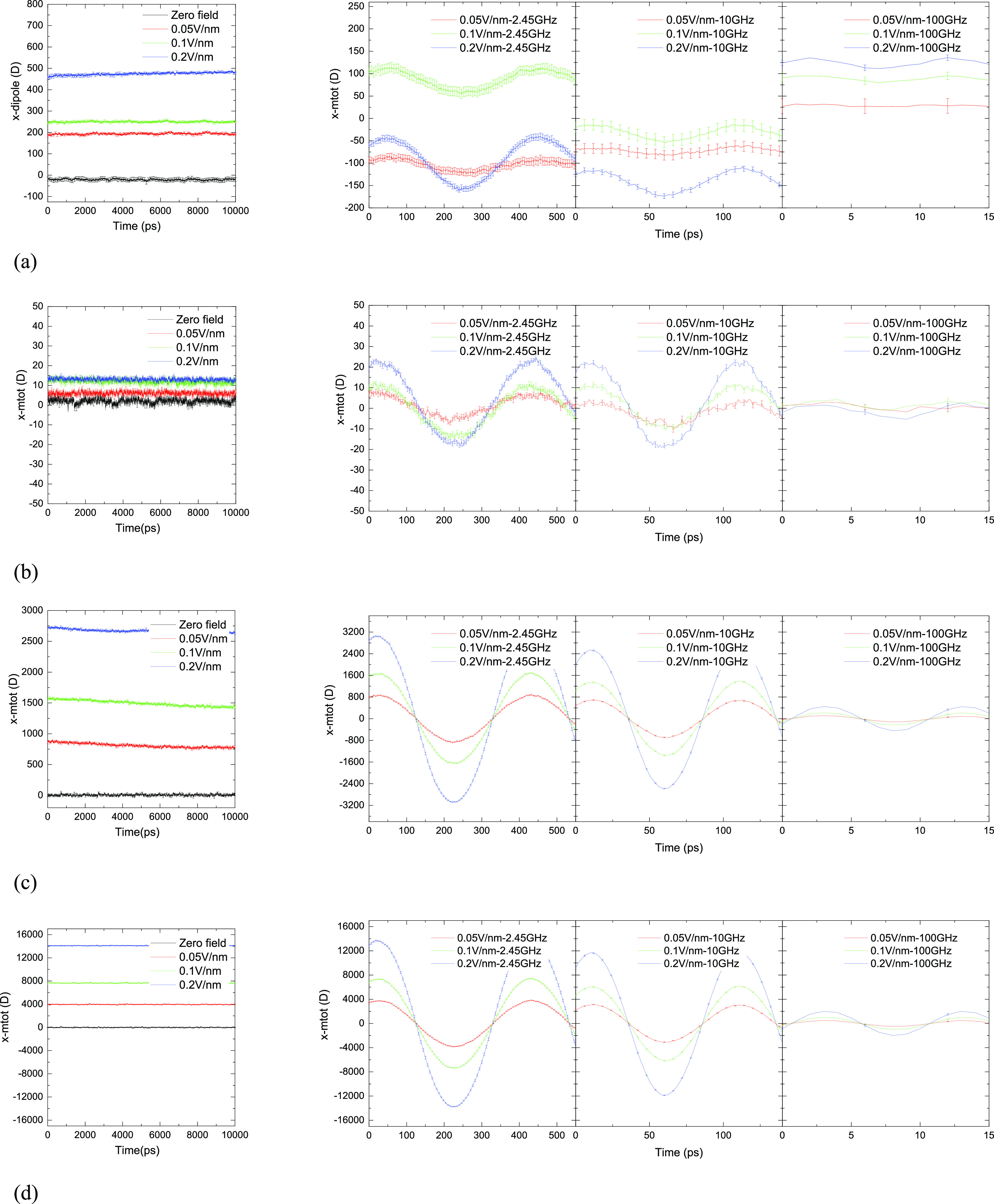
X-component of the dipole moment for (a) HEWL and water molecules in the (b) first hydration layer, the (c) second hydration layer, and (d) bulk water under different field conditions. As the average of multiple samples, the dipolar alignment process at the initial time of static-field exposure is not visible.

Figure 3 shows the collective dipole moment of the protein and hydration-layer water molecules under different field conditions. In terms of the field-alignment effect on dipoles, both the protein and water molecules present higher dipole moments numerically under the increasing intensity of the static field than under zero-field conditions. It is interesting that the maximum collective dipole moment of water molecules in the first hydration layer (*cf.*Figure 3a) is just around 15 D in magnitude, which is much less than protein (∼470 D; *cf.*Figure 3b) and water molecules in the second hydration layer (∼2700 D; *cf.*Figure 3c) and bulk (∼14,000 D; *cf.*Figure 3d). There is not a significant difference between the dipole moment of the first hydration water sub-shell under 0.01 and 0.02 V/Å static-field conditions. This can be explained by the “bounding” effect of the protein on water molecules, exerting its own intrinsic electric field in its own local hydration layer: the water molecules can form multiple hydrogen bonds with the protein, which depends on the polarity and intrinsic-field conditions in these binding sites milieux,^64,65^ and such an interplay of multiple hydrogen bonds and intrinsic electric fields can help resist the water-dipole-alignment effects of the externally applied fields. As mentioned previously, this important intrinsic field is about 2 orders of magnitude larger than external ones (*cf.*Figure 1e).

Similarly, in the case of the oscillating fields, the amplitude and frequency of the water dipole moment curve indicate the field-induced rotation and alignment of water molecules under the corresponding field. It should be noted that the amplitude of the dipole moment declines sharply in the 100 GHz fields since water molecules do not have sufficient time to approach their maximal level of dipole alignment in high-frequency fields.^60^ Furthermore, impacted by the protein limitation and insufficient response time, there is no significant cosine-wave plot of the dipole moment of water molecules in the first hydration layer in 100 GHz fields.

For HEWL itself, although previous research reported that the dipole alignment of protein cannot be observed so obviously for an intensity of 0.01 V/Å,^43,62^Figure 3, in averaging treatments for over much longer simulation time, it shows that there is a dipole-alignment effect of weaker-intensity fields over these long time scales, akin to alignment of a compass needle. However, in the case of 2.45 GHz fields, the dipole moment of HEWL for 0.01 V/Å is higher than for 0.02 V/Å, which is less consistent to the findings of English *et al.*;^43^ intriguingly, although this may hint that the direction of the applied field could impact the dipolar response of protein, the resemblance of the back-and-forth dipolar alignment response to oscillating fields to cyclic *de-facto* pendulum motion (*cf.*Figure 3) over a sufficiently large number of cycles over longer times would tend to dilute any longer-term dependence of system response to initial dipole orientation upon first field exposure.

Turning to the earlier-mentioned salt concentration and box-size effects on HEWL behavior, in which neither affected significantly the thermal motion of HEWL per se (*cf.*Figure S1a) nor dipolar orientation (*cf.*Figures S2a and S3a), but for which the 0.2 V/nm field induced substantial HEWL denaturation (*cf.*Figure S1d) and depolarization (*cf.*Figure S2d) for larger salt concentrations, we found that the underlying mechanistic reason for this stronger-field denaturation and depolarization hinged on more frequent and higher-amplitude collisions between salt ions accelerated by a stronger, static field and HEWL, which led for a greater degree of noise in the observation of a non-thermal field effect. In view of this, and taken together these elongated-box and saline-concentration observations, we conclude that the original rectangular box is sufficient for this study and can help reduce computational demands; indeed, dipole alignment along the laboratory *x*-direction can also avoid largely the effects of alignment artefacts along the laboratory *y*- and *z*-axes.

**Figure 4:**
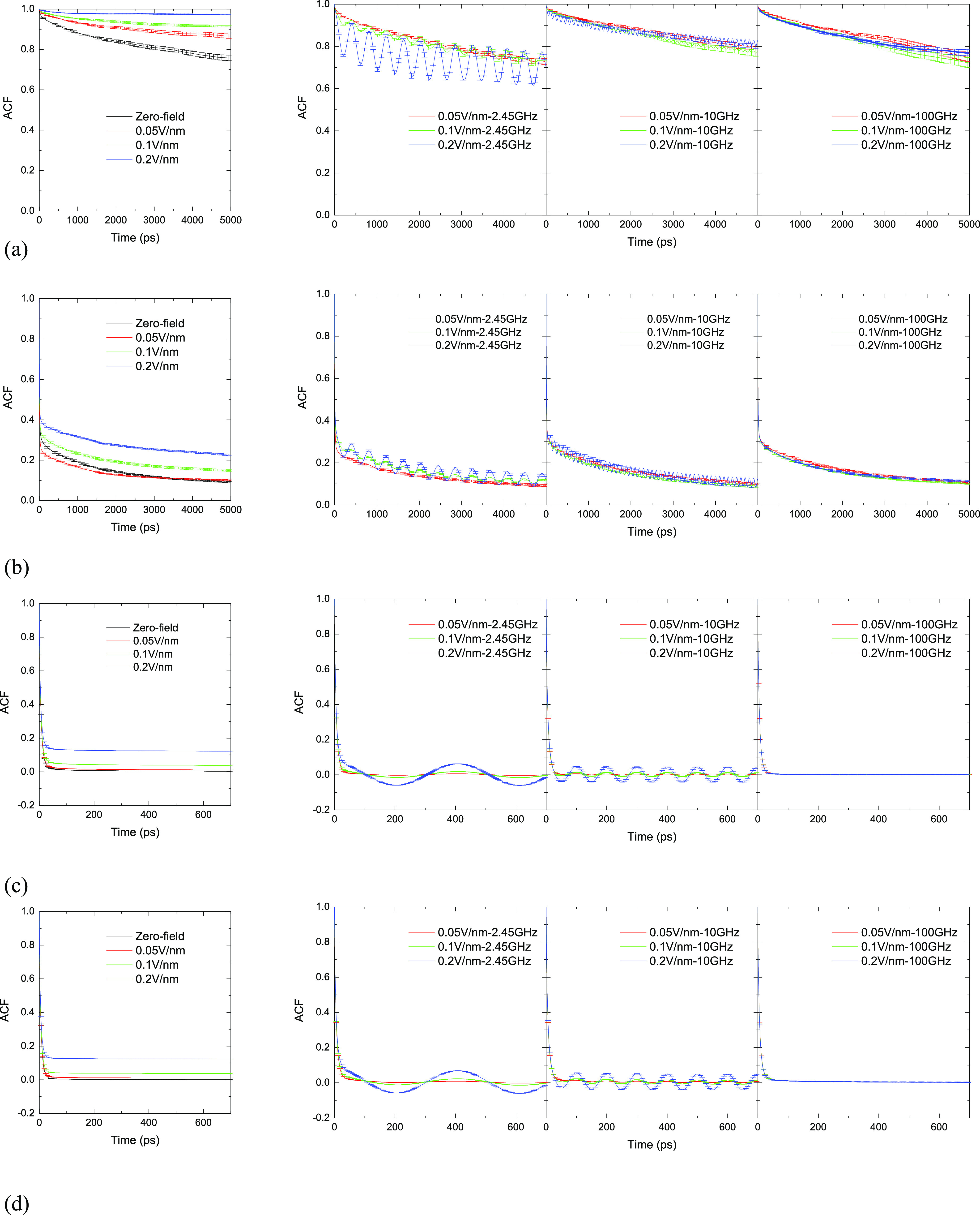
Molecular dipole moment ACFs for (a) HEWL and water molecules in the (b) first hydration sub-layer, the (c) second hydration sub-layer, and (d) bulk water under different field conditions.

The normalized ACF for the average molecular dipole moment is characterized by long-term exponential decay. Compared to water molecules in the second hydration sub-layer and the bulk (<200 ps; *cf.*Figure 4c,d), it takes more time for water molecules in the first hydration sub-layer (>5000 ps; *cf.*Figure 4b) to reach relaxation because of the large dipole moment of the protein (*cf.*Figure 4a) and intrinsic electric field in its locale (*cf.*Figure 1e), which itself prevents the surrounding water dipoles to relax rapidly. This water-dynamics effect on the hydration layer was also observed in the study of water mobility on antifreeze protein surfaces and ubiquitin,^66,67^ which reveals rather starkly the potential functional significance of this difference in dynamical behavior between the first and second hydration layer water molecules.

In the case of zero/static fields, due to dipolar alignment, dipolar relaxation decays more slowly under static fields than under the zero field—in major, existential contrast to alternating fields. On a fundamental level, water dipoles exhibit a preference to align with the field in order to optimize the dipole-field interaction energy.^65^ This field effect is not significant for the weak-field case of 0.005 V/Å and could easily be influenced by molecular motion (*e.g.*, “layer-cage” diffusion around the hydrated HEWL,^68,69^ despite minimal 90% occupation). Indeed, for the first hydration sub-layer, the ACF under 0.005 V/Å decays slightly faster than that in the zero-field case before relaxation (*cf.*Figure 4b), underpinning the local dominance of the intrinsic electric field of HEWL (*cf.*Figure 1e) in its interplay with sub-breakdown-intensity external fields (it is emphasized at this point that the very nature of the fixed-charge TIP4P/2005 model does not allow for molecular dissociation, although there is still a significance of the lowest-magnitude external field studied here, 0.005 V/Å, being dominated by the intrinsic field—for physical realism).

In the case of oscillating electric fields, the direction of the electric-field torque and force is constantly changing, which enhances the rotational motion of water molecules and makes the water molecules’ collective dipoles fluctuate around 0. External electric fields have been reported to induce a variety of field-induced anisotropies in liquid water.^70^ For instance, it was found that the field pulse leads to a strong anisotropy in hydrogen-bond orientation along with the field-induced molecular reorientation.^59^

**Figure 5:**
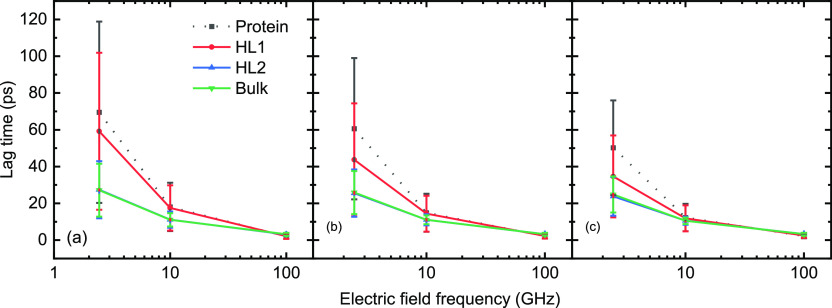
Lag time of the maximum dipole alignment for HEWL and water molecules in hydration layers relative to different external field intensities of (a) 0.05, (b) 0.1, and (c) 0.2 V/nm as a function of the field frequency.

**Figure 6:**
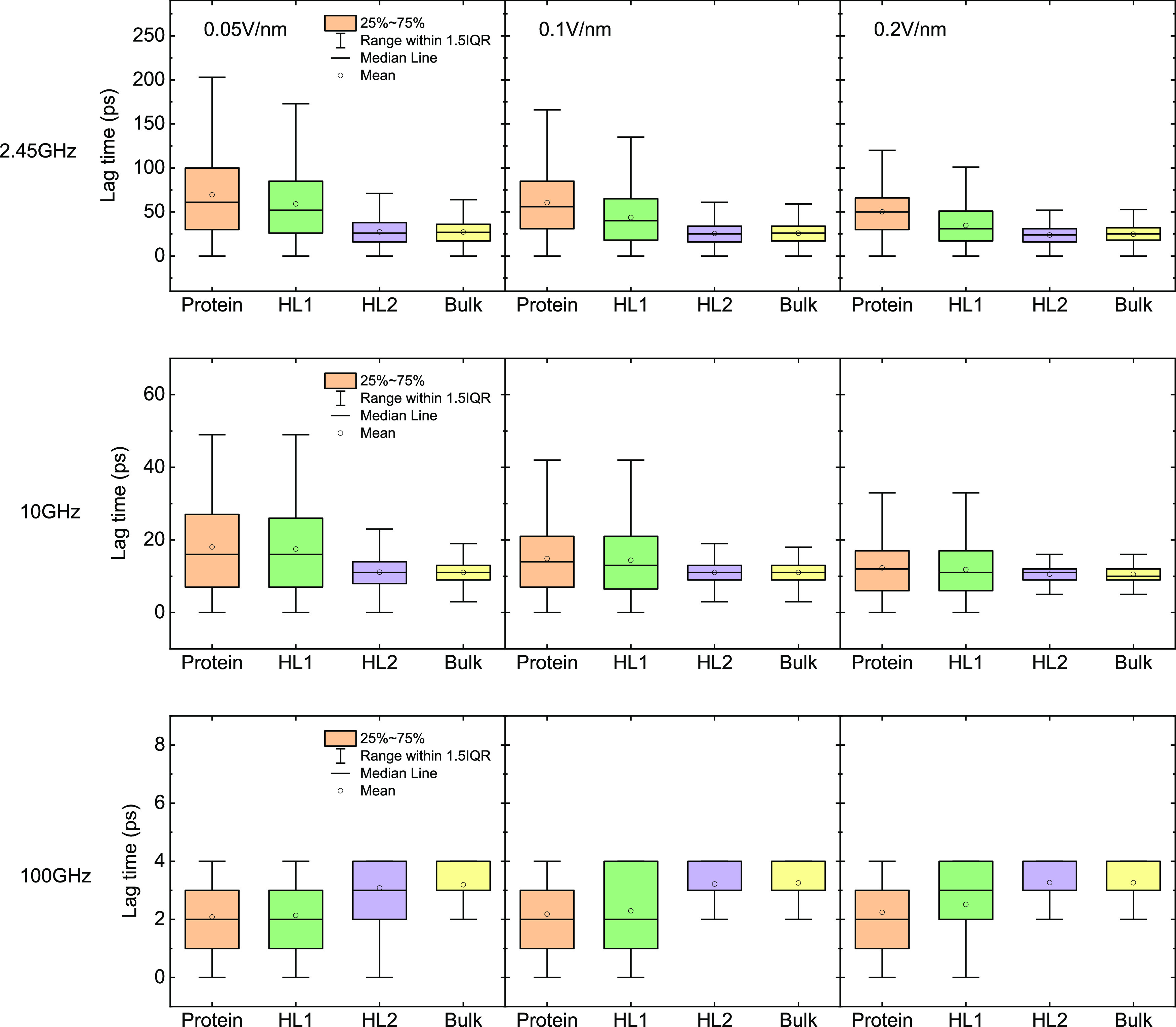
Box chart of lag time for HEWL and water molecules in hydration layers.

The rotation of protein (solute)^71^ and water molecules can be characterized by the lag time, which molecules take to reach the maximum dipole alignment under oscillating external field—lagging the original applied field.^60,72^ Compared to “slow” tumbling time scales (in nanoseconds) in the static field (*cf.*Figure 4a), the reorientation of HEWL is uncompleted in oscillating fields and, indeed, its rotational extent depends on the frequency of external fields. Figure 5 shows that the lag time of HEWL and water molecules decreases with the growth of field frequency. This is because frequent molecular reorientation in the opposite direction disrupts hydrogen bonds,^60^ leading to a more sensitive response to the external electric field.^73,74^ As a case in point, water molecules have more sensitive response to the instantaneous direction change of electric fields than carbon atoms.^75^ Thus, proteins with complex 3D conformation have variable dipolar alignment tendency in different parts, causing a wider range and longer average of lag time (*cf.*Figure 6) than the surrounding water. This effect is more significant on the first hydration sub-layer than that on the second one, and for bulk water, and it is severely weakened under 100 GHz field due to the insufficient period for complete dipole alignment. Similarly, Aparicio *et al.*(76) reported that the rotational motion of cholinium and benzoate tend to be the same under fields with frequency higher than 50 GHz. The significant deviation between the lag dipolar response of HEWL and hydration layer molecules in the lower-frequency fields (2.45 and 10 GHz) and their “homogenization” in the high-frequency case (100 GHz) indicate that the structure of protein (*e.g.*, the distribution of charged residues) and the setup of external fields dominate the dipolar response of HEWL in different field frequencies.

## 4 Conclusions

In this study, NEMD simulations were performed to determine the effect of static and oscillating electric fields on the dipolar response and hydration dynamics of solvated HEWL. The dipolar response of different hydration layers of proteins was investigated for the first time based on various frequencies and electric-field intensities. A facile trajectory-sampling method was applied to reveal clearly the dipolar-relaxation response of low-frequency electric fields based on the period of oscillating fields. Due to polarization under the electric-field exposure, both the protein and hydration water can respond sensitively to electric fields. Water molecules close to the protein in the directly adsorbed sub-layer exhibit slower dynamics than the outer hydration sub-layer, which is related to hydrogen bonds between the protein surface and adsorbed, and partly confined, water molecules. This dichotomy in rotational response caused by oscillating fields was also evident, although this difference in field response was eliminated under high-frequency fields, with such higher frequencies not allowing sufficient time for meaningful rotational response to the applied field.

More future research on water/protein hydrogen-bond dynamics would help understand the effect of the coupling mechanism of the external electric fields with the protein and its hydration layer, as well as external fields’ potential influence on modulating the biological activities of protein itself, which is not of disinterest to human health in the modern era of more ubiquitous e/m communications. Indeed, the study of how external electric fields, both static and oscillating, alter protein-tumbling dynamics^77^ is something that long-time non-equilibrium MD can tackle as microsecond sampling becomes more semi-routine.

## Supporting Information Available

Plots of rmsd of HEWL and dipole moments of HEWL and water versus time for different box sizes and salt concentrations (PDF)
