# Supplementary material for: Investigation of Dipolar Response of the Hydrated Hen-Egg White Lysozyme Complex under Externally Applied Electric Fields: Insights from Non-equilibrium Molecular Dynamics
Source: J Phys Chem B. 2022 Jan 21;126(4):858–68. doi: 10.1021/acs.jpcb.1c07096 (PMC8819654; doi:10.1021/acs.jpcb.1c07096)
Supplement: Supplementary file 1 — jp1c07096_si_001.pdf [file jp1c07096_si_001.pdf]

Investigation of Dipolar Response of Hydrated Hen-Egg White Lysozyme Complex under Externally-Applied Electric Fields: Insights from Non-Equilibrium Molecular Dynamics

HaoLun Wu[1], Mohammad Reza Ghaani[1*], Prithwish K. Nandi[1,2] and Niall J. English[1*]

1. School of Chemical & Bioprocess Engineering, University College Dublin, Belfield, Dublin 4, Ireland

2. Irish Centre for High-End Computing, Trinity Enterprise Centre, Pearse St., Dublin 2

*Corresponding authors: mohammad.ghaani@ucd.ie (MRG); niall.english@ucd.ie (NJE)

Supplemental Information

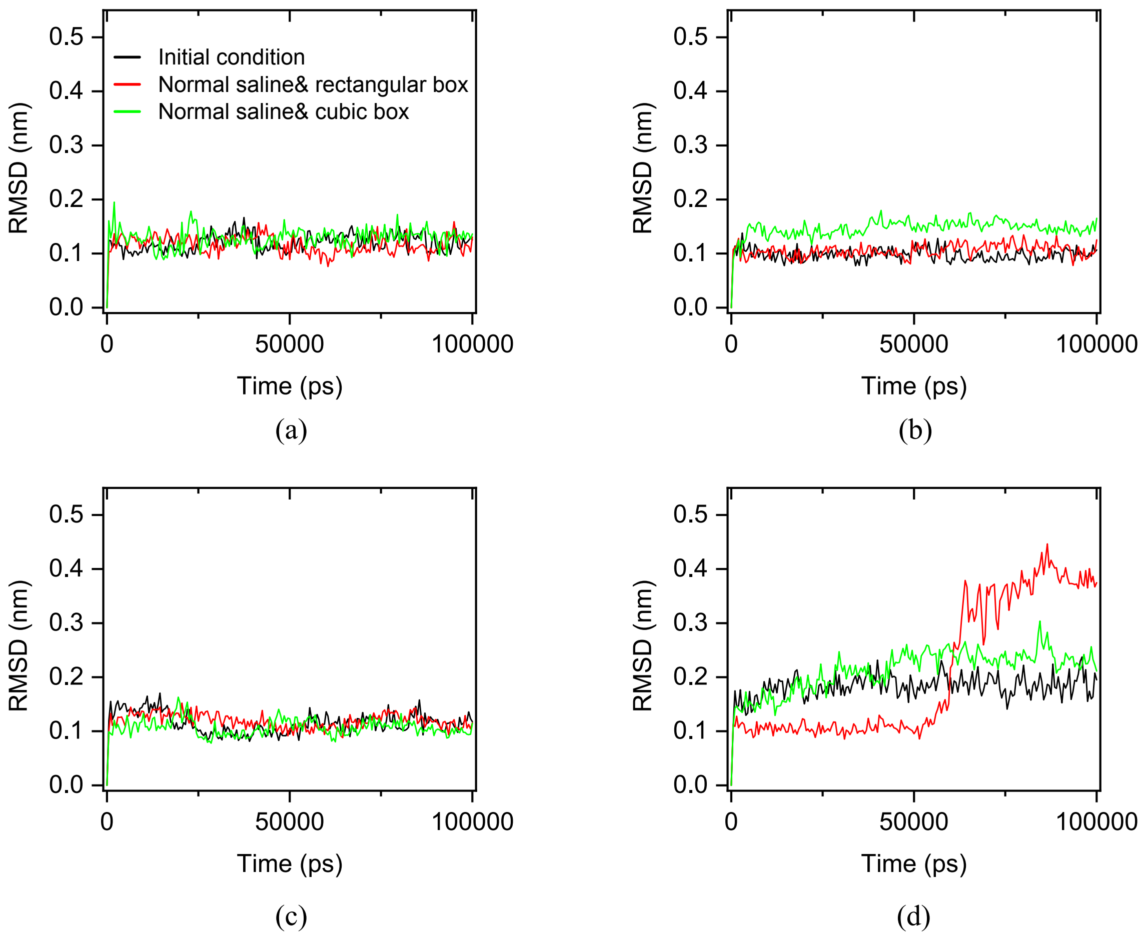

Figure S1. Root-mean-square-deviation plots as a function of time for HEWL in (a) zero field and static field with intensity of (b) 0.05 V/nm, (c) 0.1 V/nm and (d) 0.2 V/nm, where black lines represent simulations with initial parameters (159.2 × 55.2 × 61.7 Å, box size and 8 Cl^- counterions) based on the main manuscript, red lines represent simulations in an 159.2 × 55.2 × 61.7 Å box with a normal saline solution (150 mM), and green lines represent simulations in a 159.2^3 Å box with a normal saline solution. All of the same colour in this Supplementary-Information section correspond to the same setup of simulation.

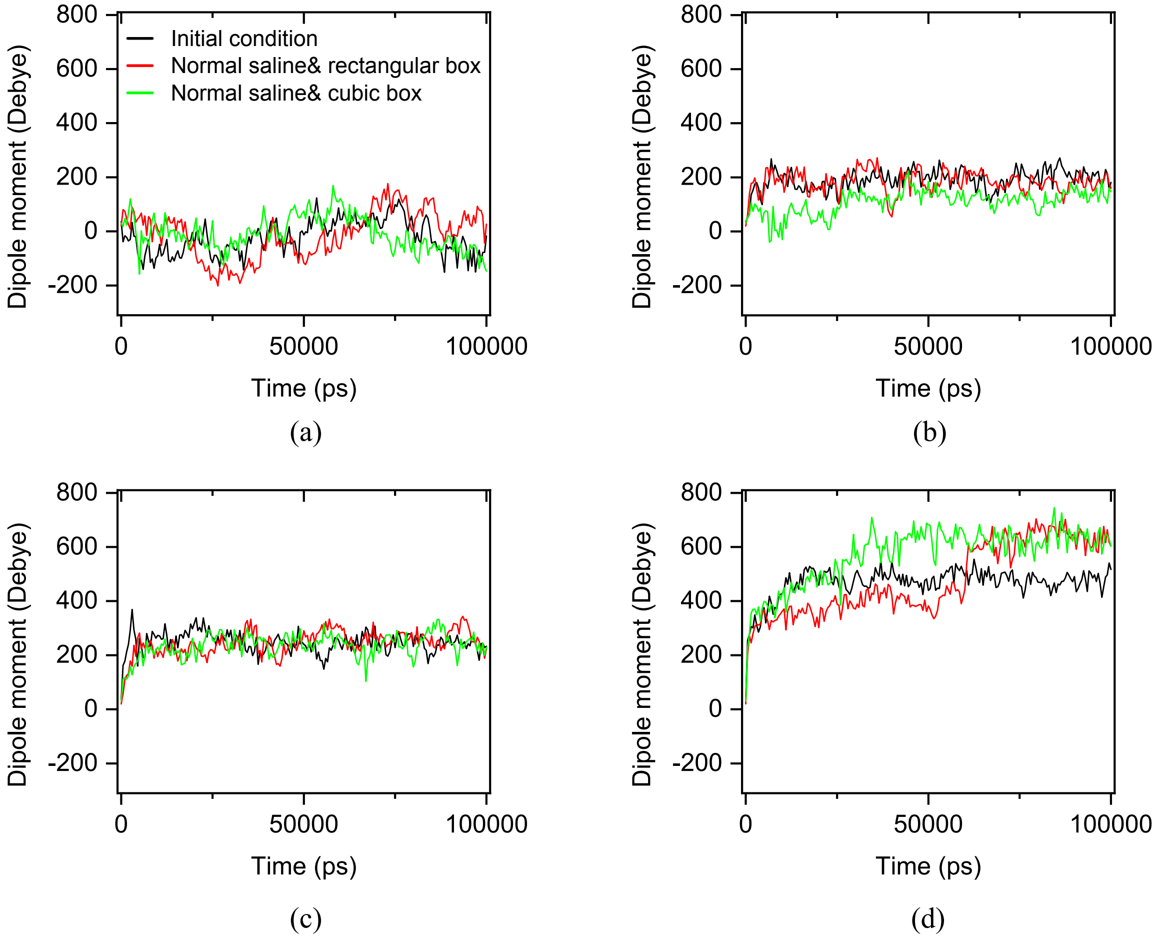

Figure S2. x-component of the dipole moment for HEWL in (a) zero field and static field with intensity of (b) 0.05 V/nm, (c) 0.1 V/nm and (d) 0.2 V/nm.

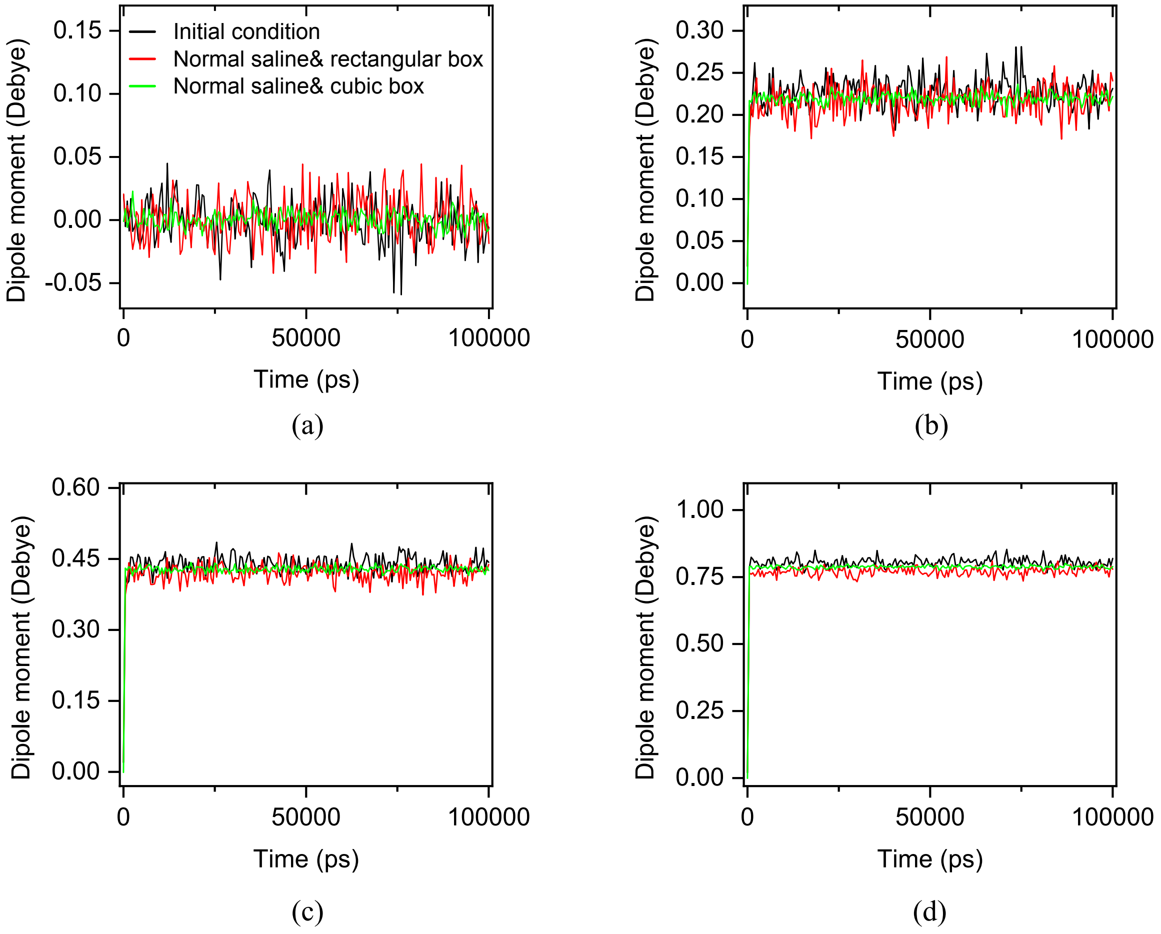

Figure S3. x-component of the average dipole moment for solvated molecules in (a) zero field and static fields with intensity of (b) 0.05 V/nm, (c) 0.1 V/nm and (d) 0.2 V/nm.
